# Association of differential gene expression with imatinib mesylate and omacetaxine mepesuccinate toxicity in lymphoblastoid cell lines

**DOI:** 10.1186/1755-8794-5-37

**Published:** 2012-08-23

**Authors:** Hemant Kulkarni, Harald H H Göring, Vincent Diego, Shelley Cole, Ken R Walder, Greg R Collier, John Blangero, Melanie A Carless

**Affiliations:** 1 Department of Genetics, Texas Biomedical Research Institute, San Antonio, TX, 78227, USA; 2 Deakin University, Geelong, VIC, Australia; 3 Barwon Biotechnology, Geelong, VIC, Australia

**Keywords:** Chronic myeloid leukemia, Microarray, Toxicity, Gene expression, Imatinib, Omacetaxine

## Abstract

**Background:**

Imatinib mesylate is currently the drug of choice to treat chronic myeloid leukemia. However, patient resistance and cytotoxicity make secondary lines of treatment, such as omacetaxine mepesuccinate, a necessity. Given that drug cytotoxicity represents a major problem during treatment, it is essential to understand the biological pathways affected to better predict poor drug response and prioritize a treatment regime.

**Methods:**

We conducted cell viability and gene expression assays to determine heritability and gene expression changes associated with imatinib and omacetaxine treatment of 55 non-cancerous lymphoblastoid cell lines, derived from 17 pedigrees. In total, 48,803 transcripts derived from Illumina Human WG-6 BeadChips were analyzed for each sample using SOLAR, whilst correcting for kinship structure.

**Results:**

Cytotoxicity within cell lines was highly heritable following imatinib treatment (h^2^ = 0.60-0.73), but not omacetaxine treatment. Cell lines treated with an IC20 dose of imatinib or omacetaxine showed differential gene expression for 956 (1.96%) and 3,892 transcripts (7.97%), respectively; 395 of these (0.8%) were significantly influenced by both imatinib and omacetaxine treatment. k-means clustering and DAVID functional annotation showed expression changes in genes related to kinase binding and vacuole-related functions following imatinib treatment, whilst expression changes in genes related to cell division and apoptosis were evident following treatment with omacetaxine. The enrichment scores for these ontologies were very high (mostly >10).

**Conclusions:**

Induction of gene expression changes related to different pathways following imatinib and omacetaxine treatment suggests that the cytotoxicity of such drugs may be differentially tolerated by individuals based on their genetic background.

## Background

Chronic myeloid leukemia (CML) represents a myeloproliferative condition characterized by a t(9;22)(q34;q11) translocation that forms the *BCR-ABL1* fusion gene [1]. The chimeric protein end-product of this gene has a dysregulated tyrosine kinase activity, which leads to disruption of several vital cellular pathways, including failure of proper apoptotic mechanisms [2,3]. The last decade has seen remarkable improvements in the treatment of CML, largely due to tyrosine kinase inhibitors like imatinib mesylate, which have now become the mainstays of CML therapy. In spite of highly encouraging reports of the protection and survival advantage conferred by imatinib treatment, [4-6] it is becoming increasingly recognized that a substantial proportion of patients acquire resistance to it (2-year incidence of resistance is ~80% in the blast phase, 40-50% in accelerated phase and 10% in the chronic phase) [7] and therefore secondary lines of treatment are essential [8]. One of the commonly used drugs in imatinib resistant cases is omacetaxine mepesuccinate, which acts by disrupting protein synthesis, degrading myeloid leukemia cell differentiation protein (MCL-1) and inducing apoptosis [9-12]. While there is a regained and renewed interest in the potential use of omacetaxine in imatinib-resistant CML cases,[1,13] full recognition of its potential is far from established.

In addition to resistance, it has been found that 3-8% of imatinib-treated subjects discontinue treatment because of the cytotoxic effects of the drug [14,15]. While the apoptotic effect of omacetaxine is known, emerging evidence suggests that imatinib can also induce cytotoxicity via apoptosis within BCR-ABL1 negative cancers through several mechanisms, including: inhibition of DNA topoisomerases [16]; increased expression of Spred2 [17]; induction of autophagy [18,19]; and induction of complement dependent pathways [20], or Bim and Bad proteins [21,22]. However, the driving molecular mechanisms involved in imatinib- and omacetaxine-induced cytotoxicity remain largely unknown.

We therefore conducted a systematic evaluation of cytotoxicity in imatinib- and omacetaxine-treated cells. In this study we tested two hypotheses. First, we conjectured that the concentration of imatinib or omacetaxine needed to inhibit growth of non-cancerous lymphoblastoid cell lines is a heritable trait. Second, we hypothesized that inhibition of cells by these drugs ensues quantifiable and characteristic changes in gene expression. Using lymphoblastoid cell lines derived from pedigreed individuals, [23] we determined the heritability of inhibitory concentrations (IC20) of both imatinib mesylate and omacetaxine mepesuccinate and identified gene expression changes associated with drug cytotoxicity.

## Methods

### Cell lines and drugs

Cell lines were derived from Epstein-Barr virus-immortalized lymphoblastoid cells of pedigreed individuals participating in the San Antonio Family Heart Study [23]. All cell lines were maintained in RPMI 1640 medium containing 2 mM L-glutamine, 1X antibiotic/antimycotic, 1 mM sodium pyruvate, 1X non-essential amino acids, 10 mM HEPES and 15% FBS (Invitrogen, Carlsbad, CA) at 37°C and 5% CO_2_. In total, 109 cell lines were assessed for imatinib mesylate cell viability and 113 cell lines were tested for omacetaxine mepesuccinate cell viability, obtained from 17 pedigrees (ranging in size from 6 to 10 individuals). Of these, 55 cell lines were further assessed for genome-wide gene expression changes in response to imatinib and omacetaxine treatment and for sensitivity of drug response studies. Cell lines were chosen based on familial relationships, allowing heritability of cell viability to be determined. Imatinib mesylate (LKT Laboratories, St Paul, MN) and omacetaxine mepesuccinate (ChemGenex, Melbourne, Australia) were solubilized in water at 10 mM and 10 μM concentrations, respectively, and stored at −20°C until used in experiments. Stock solutions were further diluted in water and then media for all assays.

### Cell viability studies

Cell viability assays were carried out in a similar manner to previous studies [24]. Cells were initially grown in 75 cm^2^ flasks, harvested and plated in triplicate into a 96 well clear-bottom plate at a density of 1×10^5^cells/ml, 180 μl per well. Cells were grown for 24 hrs and then treated with varying concentrations of either imatinib mesylate or omacetaxine mepesuccinate drug (in 20 μl volume) for 72 hrs before the addition of 20 μl alamarBlue reagent (Life Technologies, Grand Island, NY). Imatinib was added at concentrations of 2 μM, 10 μM, 50 μM, 75 μM, 100 μM, 150 μM and 200 μM and omacetaxine was added at concentrations of 5nM, 10nM, 25nM, 50nM, 75nM, 100nM and 500nM. Following the addition of alamarBlue, cells were incubated for a further 24 hours and fluorescence read at 570 nm and 600 nm using the SpectraMax 340PC 384 micro plate reader (Molecular Devices, Sunnyvale, CA). Absorbance readings for the triplicate reactions were averaged to calculate percent reduction. To calculate the percent difference in reduction of the alamarBlue reagent between treated and control cells (cell viability), the following equation was used:

(1) $$\frac{\left(117,216\times \text{test}\;\text{well}\;\text{abs}570\text{nm}\right)-\left(80,586\times \text{test}\;\text{well}\;\text{abs}600\text{nm}\right)}{\left(117,216\times \text{untreated}\;\text{well}\;\text{abs}570\text{nm}\right)-\left(80,586\times \text{untreated}\;\text{well}\;\text{abs}600\text{nm}\right)}\times 100$$

where 117,216 is the molar extinction coefficient of alamarBlue in its oxidized form at 600 nm; 80,586 is the molar extinction coefficient of alamarBlue in its oxidized form at 570 nm; and abs indicates the absorbance. GraphPad PRISM v5 software was used to plot dose response curves and determine IC20 values for both imatinib and omacetaxine for each cell line.

### Gene expression assays

Each of the 55 selected cell lines was treated with either imatinib, omacetaxine or was left untreated. Each cell line was treated for 96 hours with a concentration of drug equivalent to the IC20 value (calculated for each cell line individually); additional media containing the appropriate concentration of drug was added after the first 48 hours. A concentration equivalent to IC20 was utilized for two reasons: i) we aimed at maintaining the viability of the majority of cells to make reliable estimates of the gene expression profiles; and ii) previously published drug toxicity studies have used this cut-off in toxicological gene expression studies [25-28]. Following drug treatment, cells were collected by centrifugation at 3,000 g for 5 minutes and RNA was extracted using the RNeasy Mini Kit (Qiagen, Valencia, CA), according to the manufacturers’ instructions. RNA concentration was determined using the NanoDrop ND-1000 (ThermoScientific, Wilmington, DE) and integrity was assessed using the Bioanalyzer 2100 (Agilent Technologies, Santa Clara, CA). All samples were of high quality, having RNA integrity numbers (RIN) > 9.0. Anti-sense RNA (aRNA) was synthesized, amplified and purified from 500 ng total RNA following manufacturers’ guidelines for the Ambion MessageAmp II-Biotin Enhanced Single Round aRNA Amplification kit (Life Technologies). A total of 1.5 μg aRNA was hybridized to Illumina Human WG-6 v3 BeadChips according to manufacturers’ instructions and scanned using the Illumina® BeadArray™ 500GX Reader with Illumina® BeadScan image data acquisition software (version 2.3.0.13). To assess quality metrics of each run, several quality control procedures were implemented, including a total RNA control sample and assessment of control summary reports, which allows the user to look for variations in signal intensity, hybridization signal, background signal and the background-to-noise ratio for all samples analyzed. Illumina® BeadStudio software (version 1.5.0.34) was used for preliminary data analysis, with a standard background subtraction, to generate an output file for statistical analysis.

### Statistical analysis

**Figure 1:**
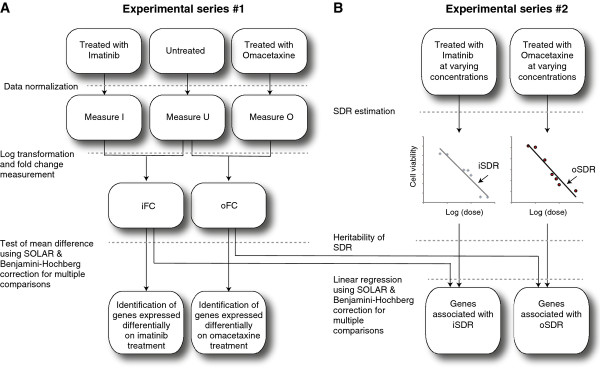
**Experimental and analytical protocol used in the study. **Experimental series 1 was undertaken to estimate the differential gene expression in 48,803 probes upon treatment with imatinib or omacetaxine. For these experiments both drugs were administered at a dose of IC20. Experimental series 2 was undertaken to measure an individual’s sensitivity to drug response (SDR) which was estimated as the slope of the regression line (arrows) between log of dose administered and cell viability. Data from these experiments were also used to estimate the IC20 values used in Experimental Series 1. Differential gene expression (iFC and oFC) was tested for statistical significance for departure from zero as well as for association with the corresponding SDR as shown. Details of the statistical methods mentioned in the figure are provided in the text.

Analyses were conducted on two sets of experimental results. Using data from Experimental Series 1 (Figure 1), we first obtained the log fold changes (differential gene expression) in response to imatinib and omacetaxine treatment (iFC and oFC, respectively). Raw signal intensity data generated in BeadStudio was normalized using quantile normalization (see Additional file 1) as described elsewhere [29]. For each probe, we then tested whether differential gene expression significantly deviated from zero using a paired Student’s *t* test. As the dataset originated from related individuals, and to correct for the consequent within-family correlations and potential kinship effects, we tested the significance of iFC and oFC using a sporadic model of the following form:

(2) $$\text{FC}=m+\beta a+e_{i}$$

where *FC* is fold change in response to a drug, *m* is average fold change attributable to the drug treatment after accounting for kinship and covariates, ***β*** is the regression coefficient vector corresponding to the covariate matrix ***a*** and *e*_*i*_ is the measurement error. The covariates used in all models were age, sex, age × sex interaction, age^2^ and age^2^ × sex interaction. The statistical significance of *m* was tested by constraining it to zero and estimating *χ*^2^(degree of freedom = 1) as twice the difference between the log-likelihood from un-constrained and constrained models. We then used the Benjamini-Hochberg method to correct for multiple comparisons based on false discovery rates. For this we used the qqvalue.ado software [30] in Stata environment. Results of these analyses were depicted as volcano plots. Lastly, we used the k-means clustering method to group the significantly differentially expressed genes (using Euclidean distance) based on the mean iFC and oFC values estimated using Equation (1).

Using data from Experimental Series 2 (Figure 1), we estimated for each probe the contribution of differential gene expression to the sensitivity of drug response (SDR), a measure of how sensitive a cell line is to changes in cell viability based on drug concentration. Sensitivity of imatinib (iSDR) and omacetaxine (oSDR) response was defined as the slope of the fitted linear regression lines that used log of dose as the independent variable and cell viability as the dependent variable. To account for within-family correlations, kinship structure and the potential heritability of SDR, we ran a polygenic model for each probe as follows:

(3) $$\text{SDR}=\mu +\beta a+g_{i}+e_{i}$$

where, *SDR* is the sensitivity of drug response, *μ* is the overall mean *SDR*, ***β*** is the regression coefficient vector corresponding to the covariate matrix ***a***, *g*_*i*_ is the polygenic effect (used to estimate the heritabilities) and *e*_*i*_ is the measurement error. In addition to the same set of covariates mentioned in Equation (1) we used the differential gene expression (iFC or oFC, generated in Equation (2)) as a covariate. We then tested the statistical significance of the regression coefficient of differential gene expression by constraining this parameter to 0 and estimating *χ*^2^(degree of freedom = 1) as twice the difference between the log-likelihood estimated from unconstrained and constrained models. Simultaneous associations of the differential gene expression with iSDR and oSDR for each probe were depicted using bivariate 95% confidence ellipses [31]. Between group differences were tested using the non-parametric Kruskal-Wallis test. We used k-means clustering analyses to infer the potential functional relevance of differentially expressed genes. For this, we used the Euclidean distance between pairs of points as the dissimilarity measure and employed the Stata program Cluster, which uses the iterative refinement approach for extracting the cluster structure to generate a pre-set number of clusters.

All genetic analyses were conducted using the SOLAR software package [32] (Version 6.3.7, Texas Biomedical Research Institute, San Antonio, TX), incorporating an additional inverse normal transformation on each trait. Statistical analyses were done using the Stata software package (Version 12.0, Stata Corp, College Station, TX). All statistical tests were conducted using global type I error rates of 0.05.

### Validation of microarray results by qPCR

To validate results from the Illumina Human WG-6 v3 BeadChips, we performed real-time reverse transcription polymerase chain reaction (quantitative PCR, qPCR) analysis on selected transcripts. We utilized *HPRT1* as an endogenous control as our microarray experiments demonstrated relative stability of gene expression across all samples when analyzed with NormFinder [33]. Taqman gene expression assays were obtained from Life Technologies (Grand Island, NY) for *BCL2L10* (Hs00368095_m1), *CTSB* (Hs00947433_m1), *HPRT1* (Hs99999909_m1), *MUL1* (Hs00226069_m1), *OIP5* (Hs00299079_m1) and *TNFAIP3* (Hs00234713_m1). The Illumina probe identifiers that corresponded to these six genes were ILMN_1749096, ILMN_1696360, ILMN_2056975, ILMN_1675055, ILMN_1759277, and ILMN_1707591, respectively. cDNA synthesis was carried out using the High Capacity cDNA Reverse Transcription Kit with RNase Inhibitor (Life Technologies), according to the manufacturers’ instructions. q-PCR analysis of each of the gene expression assays was carried out in a 10 μl reaction volume using Gene Expression Master Mix on an Applied Biosystems 7900HT instrument (Life Technologies), according to the manufacturers’ instructions. Baseline and cycle threshold (CT) were determined automatically using SDS RQ Manager software (Life Technologies). Each sample was assessed in triplicate and if the CT standard deviation for triplicate reactions was >0.3 then only duplicate reactions were assessed, if the standard deviation of duplicate reactions also exceeded 0.3, the sample was excluded from analysis. Relative quantitation was calculated using the 2^-ΔΔCT^ method in SDS RQ Manager [34]. To examine the correlation between microarray and qPCR results we used Spearman’s or Pearson’s correlation coefficients, based on the underlying distribution of the variable. In order to ensure results were directly comparable for this association, we corrected the microarray data for the *HPRT1* gene expression.

## Results

### Assessment of heritability

This study is based on lymphoblastoid cell lines derived from 55 individuals belonging to 17 pedigrees. The kinships included siblings (72), third degree relations (15) and an identical sib pair. A total of 48,803 probes, whose intensities were derived from the microarray experiments, were used for analysis. We first examined the heritability of the IC values obtained from treatment of the cell lines with imatinib (n = 109) and omacetaxine (n = 113). Although both drugs influenced cell viability in a dose-dependent manner, only the IC values for imatinib demonstrated high heritability (range: 0.60 – 0.73), IC values for omacetaxine were consistently non-heritable (Table 1).

**Table 1:** Heritability of the inhibitory concentration of experimental drugs

**Threshold (%)**	**Imatinib**			**Omacetaxine**		
	**Mean dose (x10**^**-5**^**M)**	**h**^**2**^	**P**	**Mean dose (x10**^**-8**^**M)**	**h**^**2**^	**p**
10	1.48	0.60	5.76x10^-4^	1.04	0.00	0.50
20	3.32	0.60	5.81x10^-4^	2.35	0.00	0.50
30	5.70	0.60	5.81x10^-4^	4.03	0.00	0.50
40	8.86	0.60	5.81x10^4^	6.27	0.00	0.50
50	13.29	0.60	5.73x10^-4^	9.40	0.00	0.50
60	19.03	0.73	1.92x10^-5^	14.10	0.00	0.50
70	31.00	0.60	5.73x10^-4^	21.93	0.00	0.50
80	53.14	0.60	5.81x10^-4^	37.59	0.00	0.50
90	119.60	0.60	5.74x10^-4^	84.58	0.00	0.50

h^2^, heritability.

### Differential gene expression upon treatment

**Figure 2:**
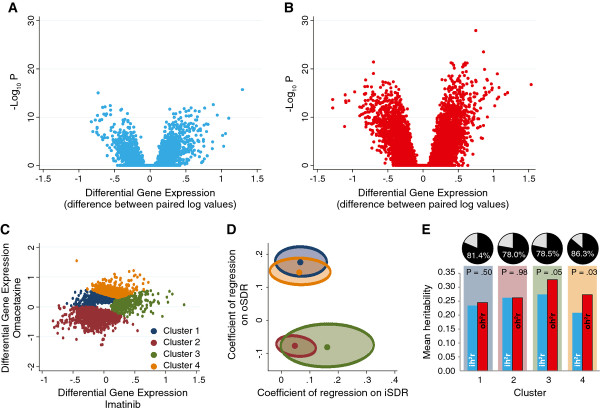
**Differential gene expression response upon treatment with imatinib or omacetaxine. (A-B) **Volcano plots depicting the extent (x-axis) and significance (y-axis) of differential gene expression for each probe set (n = 48,803) after treatment with either imatinib (A) or omacetaxine (B). **(C) **Results of k-means clustering of the statistically significant probes based on their differential gene expression in response to imatinib (x-axis) or omacetaxine (y-axis) treatment. The color codes for each cluster are indicated in the index, clusters have been referenced consistently throughout the manuscript. The lack of data towards the center of the scatter plot is due to probes that were not significant (in response to either imatinib or omacetaxine treatment), which are not shown in this figure. **(D) **Point estimates and 95% confidence ellipses for the association of differential gene expression with sensitivity of drug response (SDR). Plotted in this chart are the regression coefficients from the polygenic models (equation (2)) for the set of probes belonging to the color-coded clusters identified in panel C. **(E) **Heritability of differential gene expression. The data are shown separately for probe sets belonging to each cluster identified in panel C. Pie charts at the top demonstrate the proportion of probe sets within the corresponding cluster that showed a heritability value exceeding zero for differential gene expression in response to imatinib as well as omacetaxine. The bar charts show the mean heritability of imatinib (blue) and omacetaxine (red) response for genes within each cluster. For reference, color-coded background is shown on the chart corresponding to the clusters. Within each cluster, the difference in the mean heritability for imatinib and omacetaxine response was assessed using a paired Student’s *t *test, the result of which is shown as the p-value at the top of the bars. ih2r, heritability of differential gene expression in response to imatinib; oh2r, heritability of differential gene expression in response to omacetaxine.

We next examined whether there was a significant difference in gene expression following treatment with either imatinib or omacetaxine at an IC20 equivalent dose. For imatinib, the IC20 values ranged from 8.7×10^-6^M to 8.7×10^-5^ M and for omacetaxine, the IC20 values ranged from 1.0×10^-8^ M to 2.2×10^-7^ M. Volcano plots (Figure 2A and 2B) indicate numerous statistically significant differences in gene expression upon treatment with both drugs. The mean enrichment score, defined as the average –log_10_ p-value, for all 48,803 probes was 0.09 (SD 0.66) after imatinib treatment and 0.46 (SD 1.85) after omacetaxine treatment; indicating a statistically significant difference in gene expression induction between imatinib and omacetaxine treatment (paired Student’s t = 44.886, degrees of freedom = 48,802, tails = 2, p <1x10^-22^). Following imatinib treatment, 956 probes (1.96%) demonstrated differential gene expression, whilst 3,892 probes (7.97%) showed differential gene expression following omacetaxine treatment (FDR corrected p-values <0.05). A total of 395 of these probes (0.8%) were significantly influenced by both imatinib and omacetaxine treatment. We reduced the dimensionality of the dataset by using k-means clustering to generate four non-overlapping clusters, which highlighted those probes showing differential gene expression following treatment with imatinib or omacetaxine (Figure 2C).

Probes within cluster 1 (n = 1,378, 30.95%) were mildly down regulated by imatinib and mildly up regulated by omacetaxine; those in cluster 2 (n = 2,005, 45.03%) were down regulated by both imatinib and omacetaxine; those in cluster 3 (n = 428, 9.61%) were mainly up regulated by imatinib and those in cluster 4 (n = 642, 14.42%) were mainly up regulated by omacetaxine. Corroborating these conceptual inferences, the mean iFC values and the enrichment scores [mean -log_10_ (FDR-corrected p-value)] for probes in the four clusters were −0.02 and 0.35 for cluster 1; -0.08 and 0.63 for cluster 2; 0.36 and 4.20 for cluster 3; and 0.13 and 0.67 for cluster 4. The mean oFC values and enrichment scores for these clusters were 0.26 and 4.31 for cluster 1; -0.35 and 5.05 for cluster 2; 0.10 and 0.89 for cluster 3 and 0.5 and 8.44 for cluster 4.

### Functional annotation of identified genes

We next considered whether these unsupervised and unbiased clusters of probes indicated any underlying functional similarities. First, we used the DAVID® functional annotation clustering tool [35,36] to map probes that showed significant differential gene expression onto Gene Ontology (GO) terms (Table 2); 83% – 89% of the significantly identified probes in various clusters were mapped (overall mapped fraction 85.4%). The full functional annotation of all significant probes is provided in Additional file 2 for each cluster (see Additional file 2). Interestingly, the top 5 functional annotation groups that mapped onto the four clusters identified in this study varied substantially with differing enrichment scores. For instance, omacetaxine treatment appeared consistent with reduced cell division and increased apoptosis while increased kinase binding and vacuole-related functions were associated with an up regulation in response to imatinib.

**Table 2:** Concordance of functional annotation cluster (using DAVID®) with the four clusters identified based on differential gene expression

**Description**	**Cluster 1**	**Cluster 2**	**Cluster 3**	**Cluster 4**
Number of genes				
Total	1378	2005	428	642
Identified as human by DAVID	1332	1932	416	617
Mapped by DAVID with GO terms	1187 (86.1%)	1704 (85.0%)	380 (88.8%)	532 (82.9%)
Functional cluster rank (enrichment score*)				
1	Mitochondria	Mitosis	Vacuole	Anti-apoptosis
	(12.08)	(24.40)	(10.18)	(2.72)
2	Ribosomes	Kinetochore	Endocytosis	Kinase binding
	(7.72)	(16.34)	(2.82)	(2.67)
3	Catabolism	Intracellular organelle	Cell fraction	Apoptosis regulation
	(6.53)	(12.53)	(2.56)	(2.59)
4	Intracellular organelle	DNA Replication	Metal ion binding	Intracellular organelle
	(4.80)	(11.96)	(2.37)	(2.25)
5	Ubiquitination	DNA Repair	Lysosome	Metal ion binding
	(4.10)	(11.85)	(1.86)	(2.05) and
				Tyrosine kinase activity
				(2.05)

*DAVID* Database for Annotation, Visualization and Integrated Discovery; *GO* gene ontology. enrichment scores are the average –log_10_ p-values for the genes loading onto the specified functional cluster.

Second, we examined whether each significantly up regulated probe was associated with iSDR and oSDR. We found that probes in clusters 1 and 4 did not differ from each other with respect to their relation with iSDR and oSDR as indicated by largely overlapping 95% confidence ellipses (Figure 2D). Similarly, the confidence ellipses of clusters 2 and 3 overlapped to a large extent. However, the 95% confidence ellipses of clusters 1 and 4 differed significantly from those of clusters 2 and 3. The 95% confidence ellipses for clusters 1 and 4 (which indicate probes that were significantly up regulated by omacetaxine but had variable response to imatinib) were more positively correlated with oSDR than the remaining clusters. Imatinib treatment did not appear to make a statistically significant difference in association with iSDR. In effect, the probes could be clustered into only two discrete groups based on their regression on to iSDR and oSDR.

Third, using polygenic models, we found that differential gene expression in response to imatinib (ih^2^r) and omacetaxine (oh^2^r) for the majority of probes had measurable (that is exceeded zero) heritability (Figure 2E). In probes with a measurable heritability, the estimates of oh^2^r were significantly higher than those for ih^2^r for those probes grouped in clusters 3 (p = 0.05) and 4 (p = 0.03). These observations indicate that gene response to omacetaxine was more heritable than gene response to imatinib, especially in clusters 3 and 4.

Previously, we had identified 750 (3.82%) *cis*-regulated genes (*cis*-LOD > 3) within a subset of 19,648 transcripts whose expression was measured in lymphocytes of individuals within the San Antonio Family Heart Study [29]. Of these *cis*-regulated transcripts, we identified 41 (5.73%), 57 (5.35%), 8 (3.51%) and 14 (4.42%) that were present in clusters 1–4, respectively. The number of *cis*-regulated transcripts was significantly higher in clusters 1 (*χ*^2^ = 6.81, p = 0.009) and 2 (*χ*^2^ = 6.54, p = 0.011), as compared to the overall expected proportion of *cis*-regulated genes (3.82% as mentioned above). Genes in clusters 3 and 4 did not demonstrate a systematic difference in the proportion of *cis*-regulated genes (p = 0.810 and 0.587, respectively). Thus, the genes that were down regulated by imatinib treatment (clusters 1 and 2) contained a significantly higher proportion of *cis*-regulated genes.

### Validation of microarray results

**Figure 3:**
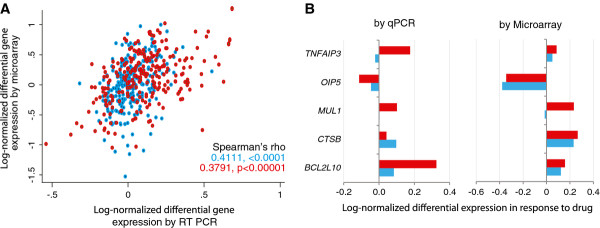
**Validation of the microarray results by qPCR. (A) **Scatter plot of the differential gene expression in response to imatinib (blue) and omacetaxine (red). Both axes show log-transformed differential gene expression. Results are for all five genes that were assayed by microarray and qPCR. Since qPCR used *HPRT1 *as the normalizer gene, the microarray based results are shown after correction for *HPRT1 *expression. **(B) **Mean differential gene expression for the five selected genes by qPCR (left panel) and microarray (right panel) in response to imatinib (blue bars) and omacetaxine (red bars) treatment. The bars represent log-transformed differential gene expression values.

We chose five representative genes within the four clusters identified from the microarray results and conducted qPCR assays to validate the differential expression of these genes in response to imatinib and omacetaxine treatment. Amplification plots for a representative sample for all five genes are shown in Supplementary Figure 2 (see Additional file 3). Comparison between the microarray and qPCR results indicated a statistically significant positive correlation (Figure 3A), showing that gene expression results derived from the two assays varied in similar directions. However, the gene-specific correlations shown in Supplementary Table 2 (see Additional file 4) demonstrate variability of correlation across genes. Although not always significant, the gene expression changes seen between our qPCR and microarray results were typically correlated. To better examine whether the two assays demonstrate similar patterns of gene expression changes, we estimated the mean differential expression of *TNFAIP3*, *OIP5*, *MUL1*, *CTSB* and *BCL2L10* derived from each method with the goal of replicating the patterns of differential expression. We found that the direction of change in gene expression was the same between the two assays for all five genes following omacetaxine treatment, but only 3 of the five genes following imatinib treatment (Figure 3B); qPCR results for *MUL1* and *TNFAIP3* did not corroborate the microarray results for imatinib treatment. The Pearson’s correlation coefficient in the mean differential gene expression estimated by microarray and qPCR was 0.63. Our qPCR results therefore demonstrate a direction of effect correlation with the results from microarray experiments.

### Differential gene expression and sensitivity of response

**Figure 4:**
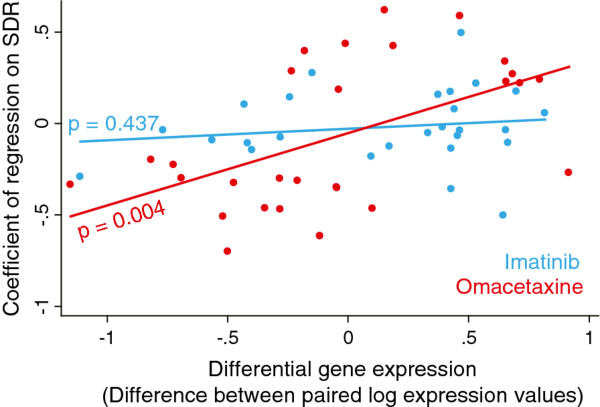
**Association between differential gene expression and the SDR in the 34 significant probes. **Charts show scatter plots and correspondingly color-coded regression lines. The p-values shown along the regression lines are for a test of the null hypothesis: regression coefficient = 0.

We considered the comparative strength of association of differential gene expression (iFC or oFC) with the sensitivity of drug response (iSDR or oSDR, respectively). For this we selected all probes which showed a FDR-corrected statistically significant correlation between FC and SDR values. We identified 21 genes that showed a significant association between iFC and iSDR and 13 genes that showed a significant association between oFC and oSDR. We annotated these genes using the GeneGO tool (Table 3). Several interesting candidate genes, such as *STAT1, GPS1, YY1, CYFIP2, and CYP1B1, PRPF19, KNTC1, GP2* and *EDN1*, which are known to partake in important signaling pathways, were identified. We also found that there was a significant positive correlation between oFC and oSDR (p = 0.004) but not for iFC and iSDR (Figure 4).

**Table 3:** Annotation of the 34 genes that were differentially expressed upon treatment as well as associated with SDR

**Probe ID**	**Gene symbol**	**Gene name**	**Cluster**	**iFC**	**oFC**	**Pathways**
ILMN_1691364	*STAT1*	Signal transducer and activator of transcription 1, 91 kDa	1	−0.1071	0.2270	IFNα/ß/Γ; oncostatin M, Thrombopoetin; Angiopoetin, PDGF; IL-1/IL-9/IL-22;/IL23; EGFR; p53; SUMO-1; Leptin
ILMN_1700028	*C9orf156*	Chromosome 9 open reading frame 156	1	−0.0526	0.2686	
ILMN_1747771	*MAEA*	Macrophage erythroblast attacher	1	−0.0370	0.2202	
ILMN_1751803	*LSM10*	LSM10, U7 small nuclear RNA associated	1	−0.0924	0.1841	
ILMN_1770892	*YY1*	YY1 transcription factor	1	0.0556	0.2401	TGF-ß signaling, NOTCH signaling, p53 signaling
ILMN_2258268	*GLRX2*	Glutaredoxin 2	1	−0.1088	0.2979	
ILMN_2309228	*GPS1*	G protein pathway suppressor 1	1	0.0364	0.3378	
ILMN_1659753	*LAMP2*	Lysosomal-associated membrane protein 2	2	0.0400	−0.2695	
ILMN_1677691	*GP2*	Glycoprotein 2 (zymogen granule membrane)	2	0.0012	−0.3267	Integrin inside-out signaling
ILMN_1682775	*EDN1*	Endothelin 1	2	−0.5040	−0.4670	Leptin signaling via JAK/STAT & MAPK, EGFR transactivation, IL-1 signaling, EDRNA signaling
ILMN_1695058	*SLC38A5*	Solute carrier family 38, member 5	2	−0.1257	−0.2268	
ILMN_1703906	*HJURP*	Holliday junction recognition protein	2	−0.0682	−0.6162	
ILMN_1707591	*TNIP3*	TNFAIP3 interacting protein 3	2	−0.3585	−0.5433	
ILMN_1713952	*C1orf106*	Chromosome 1 open reading frame 106	2	−0.0368	−0.3012	
ILMN_1732516	*KNTC1*	Kinetochore associated 1	2	−0.0216	−0.3534	Spindle assembly
ILMN_1769545	*PRPF19*	PRP19/PSO4 pre-mRNA processing factor 19 homolog (S. cerevisiae)	2	−0.0770	−0.3237	NOTCH signaling
ILMN_1774077	*GBP2*	Guanylate binding protein 2, interferon-inducible	2	−0.0388	−0.4636	
ILMN_1849494	*EFR3B*	EFR3 homolog B (S. cerevisiae)	2	−0.0256	−0.4698	
ILMN_1868655	*AI916641*		2	−0.1451	−0.4635	
ILMN_1898692	*BI254341*		2	−0.2912	−0.3491	
ILMN_1912827	*CB157495*		2	−0.1382	−0.5097	
ILMN_2103685	*DEPDC1B*	DEP domain containing 1B	2	−0.1807	−0.7007	
ILMN_2230162	*FLJ44124*	Hypothetical LOC641737	2	0.0233	−0.3135	
ILMN_2336595	*ACSS2*	Acyl-CoA synthetase short-chain family member 2	2	0.1570	−0.5157	
ILMN_2384544	*ADAM15*	ADAM metallopeptidase domain 15	2	0.2186	−0.3365	
ILMN_1677200	*CYFIP2*	Cytoplasmic FMR1 interacting protein 2	3	0.3287	−0.2998	G-protein signaling RAC1
ILMN_1724437	*GCAT*	Glycine C-acetyltransferase (2-amino-3- ketobutyrate coenzyme A ligase)	3	0.2763	−0.1991	
ILMN_1789436	*AK091207*		3	0.4950	0.2848	
ILMN_2392352	*CTPS2*	CTP synthase II	3	0.1718	0.0349	
ILMN_1664798	*GRHPR*	Glyoxylate reductase/hydroxypyruvate reductase	4	0.1026	0.4346	
ILMN_1693338	*CYP1B1*	Cytochrome P450, family 1, subfamily B, polypeptide 1	4	0.1427	0.4242	Benzopyrene, estradiol and retinol metabolism
ILMN_1764361	*DUSP16*	Dual specificity phosphatase 16	4	0.2176	0.6187	p53; Erk
ILMN_1779428	*C12orf68*	Chromosome 12 open reading frame 68	4	0.0770	0.3965	
ILMN_1794956	*BBS9*	Bardet-Biedl syndrome 9	4	0.1743	0.5875	

## Discussion

Our study made three novel and interesting observations. First, inhibitory concentration values following treatment with imatinib were highly heritable, although this was not seen for treatment with omacetaxine. This finding implies that in a clinical scenario, determination of a toxic dose of omacetaxine would not need to consider potential familial and heritable influences of drug cytotoxicity. Second, differential gene expression in response to omacetaxine was stronger and more widespread in the genome than for imatinib. Third, the differential gene expression after omacetaxine exposure was a significant determinant of an individual’s sensitivity of drug response (defined as the slope of regression line between cell viability and log of drug concentration) but a similar association was not found in the context of imatinib treatment. Together, this suggests that while the dose required to induce cell death is not heritable for omacetaxine, an individual’s sensitivity of genomic response to a fixed dose of this drug is heritable. In contrast, while imatinib toxicity was highly heritable, the extent of genomic response was more subdued and was not predictive of the sensitivity of an individual’s response to a fixed dose of imatinib. These findings not only demonstrate the differences between the toxicity profiles of imatinib and omacetaxine, but also point towards the possibility of successfully using gene-agnostic drugs as a secondary line of treatment in cases of imatinib resistance.

Functional annotation confirmed results from previous studies that identified kinase binding, apoptosis and vacuole/lysosome related genes as targets of imatinib treatment [37,38]. It is noteworthy that a recent genome-wide association study in yeast identified the vacuolar proton transporter ATPase (V-ATPase) protein, which maintains vacuolar pH, as an important target of imatinib action [39]. Also, our results indicate that JAK/STAT, MAPK, Akt, Leptin and NOTCH signaling pathways may be perturbed by both imatinib and omacetaxine (Table 3), findings that have been observed in previous studies [40-46]. However, the fact that these pathways were implicated in the list of the 34 genes associated with both differential gene expression and sensitivity to drug response is a novel finding.

### Limitations

In addition to the limitations implicit in any microarray study, our study has further limitations. First, this study was conducted in EBV-transformed lymphoblastoid cell lines and therefore it should be noted that the differential gene expression being ascribed to drug treatment are on the background of an EBV transformation, which may not truly reflect clinical changes. It is noteworthy that imatinib can inhibit the virus-specific proliferation of CD8^+^ T cells such that there are varying degrees of this inhibitory effect to peptides derived from cytomegalovirus or EBV [47]. It has also been reported that one of the manifestations of imatinib resistance is EBV-positive cutaneous B-cell lymphoproliferative disease [48,49]. Given that these cell lines were all generated in an identical manner, we do not anticipate that all findings within this study would be a consequence of such manifestations. Consistent differential gene expression changes shown across all cell lines are likely to represent true gene expression changes, unrelated to the effect of EBV transformation, however caution should be taken in the interpretation of these results. Evidence demonstrating an interaction between omacetaxine and EBV is currently not available.

Second, the microarray studies were conducted by treating cell lines for 4 days with a preselected concentration equivalent to the IC20 dose for each drug (to avoid excessive cell death). It is possible that clinical gene expression changes would be different from those identified in this study, dependent on the dose chosen and length of treatment. Although our study does not permit interpretation about dose-related differential gene expression, we demonstrate here, examples of pathways that are likely to be altered by imatinib or omacetaxine treatment, many of which have very plausible biological roles in such drug cytotoxicity.

Third, even though the correlations between results of qPCR and microarray estimates can be considered to be modest, these correlations are consistent with those reported in literature [50]. Indeed, several sources of variation like degree of differential expression, spot intensity, array averaging and filtering, probe design, binding sites and variances in amplification methodologies prevent a direct validation of microarray results by qPCR. Still, our qPCR results showed moderate concordance with the microarray assay.

We performed this study to gain a better understanding of the effects of imatinib and omacetaxine treatment on non-cancerous cells in order to identify pathways that might be consistent with drug cytotoxicity. Our results can therefore only be interpreted as potential leads for further research into the cytotoxic effects of two drugs commonly used to treat CML.

## Conclusions

We have identified genetic signatures of imatinib and omacetaxine toxicity in non-cancerous cell lines derived from closely related individuals. Identification of the pathways induced by either imatinib or omacetaxine treatment on non-cancerous cell lines may lead to the possibility of early detection of subjects who might be more prone to develop drug cytotoxicity. To our knowledge such a microarray study has not been conducted thus far. Future research should focus on the candidate genes identified in this study as potential determinants of drug toxicity in the treatment of CML. Investigation of such gene expression profiles in CML patients would be particularly interesting, and may be useful in determining a cost/benefit ratio of a treatment regime.

## Abbreviations

CML: Chronic myeloid leukemia; MCL-1: Myeloid leukemia cell differentiation protein; FC: Fold change; SDR: Sensitivity of drug response; IC: Inhibitory concentration; FDR: False discovery rate; GO: Gene ontology; qPCR: Quantitative polymerase chain reaction.

## Competing Interests

The authors declare that they have no competing interests.

## Authors’ contributions

JB, KRW, GRC and MAC designed the study. SC was responsible for overseeing the generation of cell lines. MAC performed all molecular analyses HK, VD, HHHG and JB performed statistical analysis. HK, MAC and JB wrote the manuscript. All coauthors read and approved the final manuscript.

## Pre-publication history

The pre-publication history for this paper can be accessed here:

http://www.biomedcentral.com/1755-8794/5/37/prepub

## Supplementary Material

Additional file 1 **Figure S1. **Quantile normalization of gene expression data. Click here for file

Additional file 2 **Table S1. **Clusterwise functional annotation of the significant probes. Click here for file

Additional file 3 **Figure S3. **Representative amplification plots from RT-PCR for selected genes. Click here for file

Additional file 4 **Table S2. **Gene-specific correlation between the results of q-PCR and microarray. Click here for file
