# Supplementary material for: Association of differential gene expression with imatinib mesylate and omacetaxine mepesuccinate toxicity in lymphoblastoid cell lines
Source: BMC Med Genomics. 2012 Aug 23;5:37. doi: 10.1186/1755-8794-5-37 (PMC3483163; doi:10.1186/1755-8794-5-37)
Supplement: Additional file 1 — Figure S1. Quantile normalization of gene expression data. [file 1755-8794-5-37-S1.doc]

**Supplementary Figure 1**


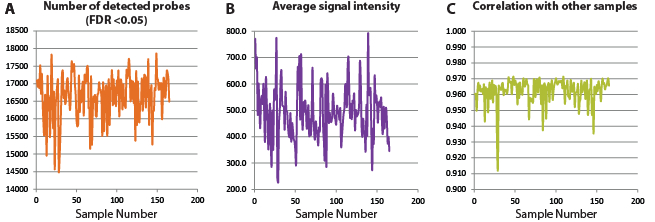


**Supplementary Figure 1. Quantile normalization of gene expression data**. **(A)** Number of probes detected to be significant (based on detection p value and a false discovery rate < 0.05) for each sample (n=165); **(B)** Average signal intensity for each sample ; and **(C)** Pearson’s correlation coefficient for overall correlation of a sample with the remaining samples.
