# Supplementary material for: Association of differential gene expression with imatinib mesylate and omacetaxine mepesuccinate toxicity in lymphoblastoid cell lines
Source: BMC Med Genomics. 2012 Aug 23;5:37. doi: 10.1186/1755-8794-5-37 (PMC3483163; doi:10.1186/1755-8794-5-37)

**Supplementary Figure 2: Amplification plots for all the genes in a representative sample**

1. *HPRT1* – the normalizer gene


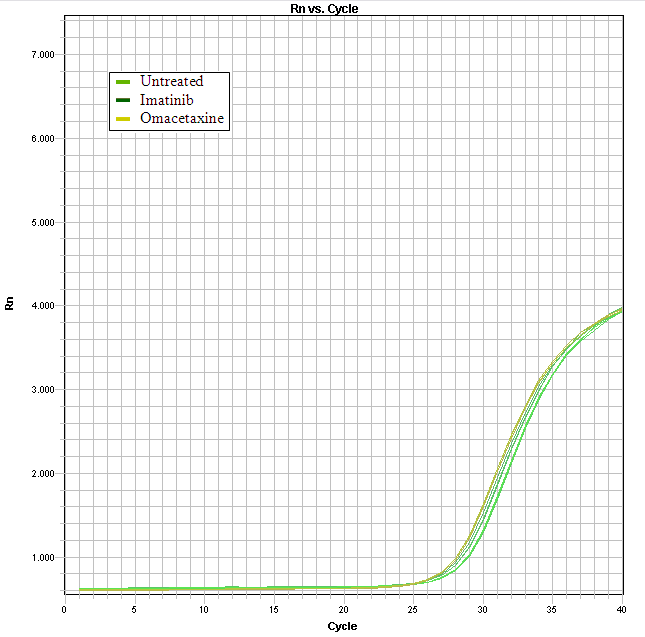


1. *TNIP3*


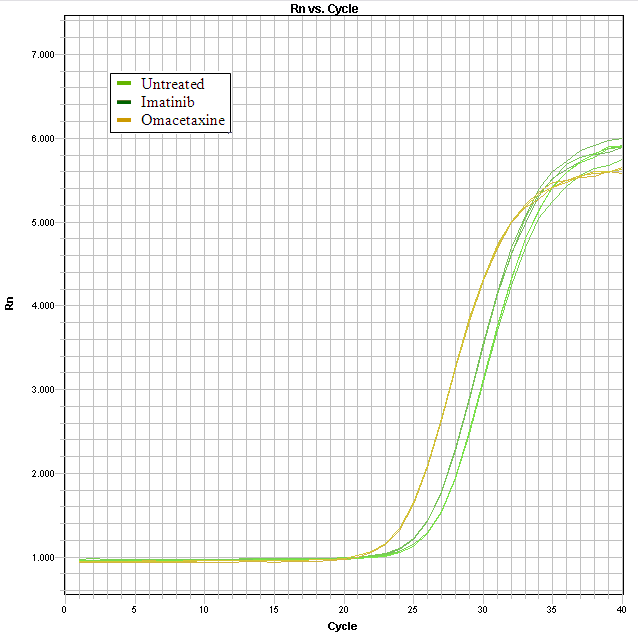


1. *OIP5*


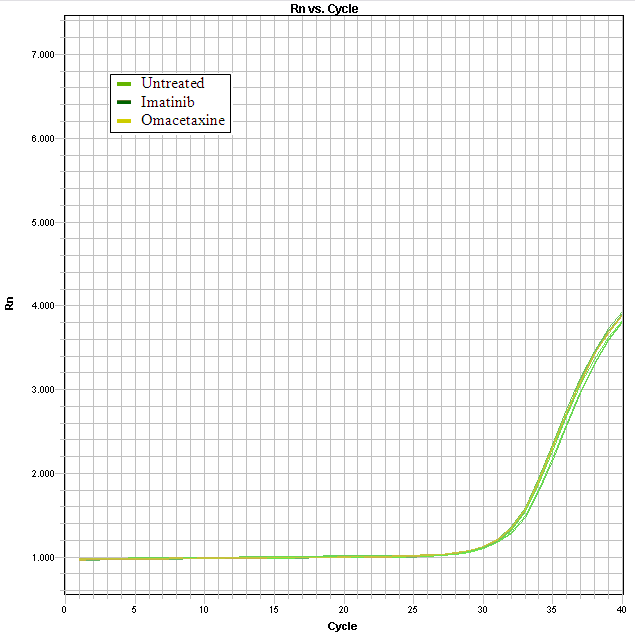


1. *MUL1*


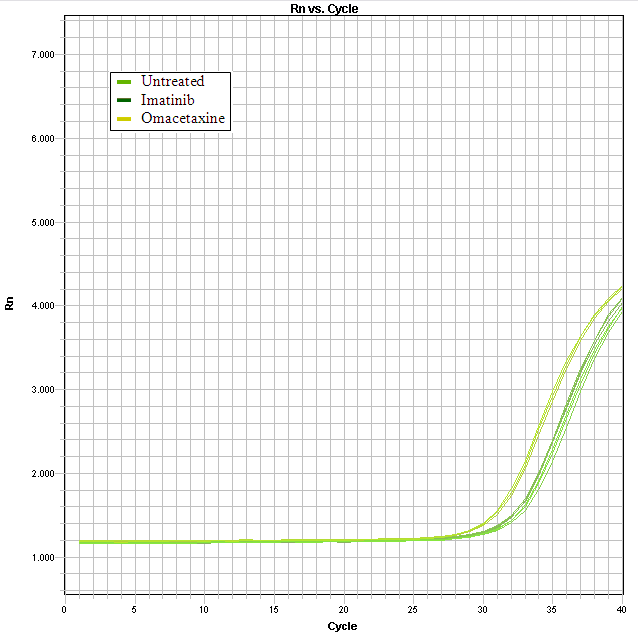


1. *CTSB*


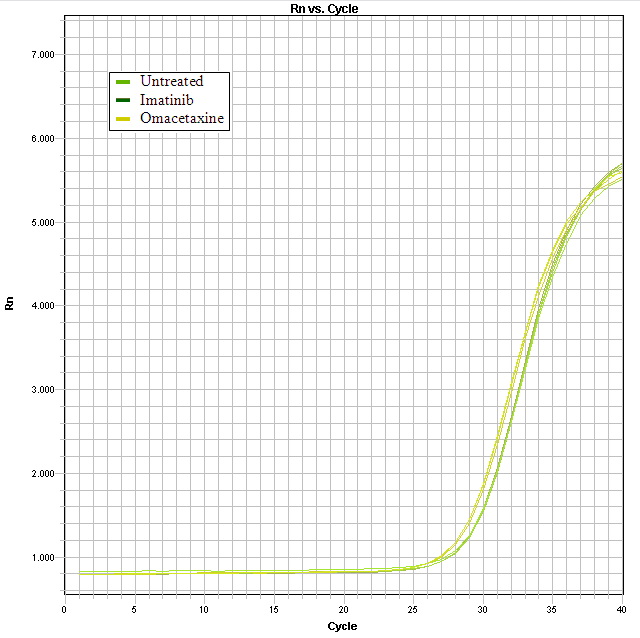


1. *BCL2L10*


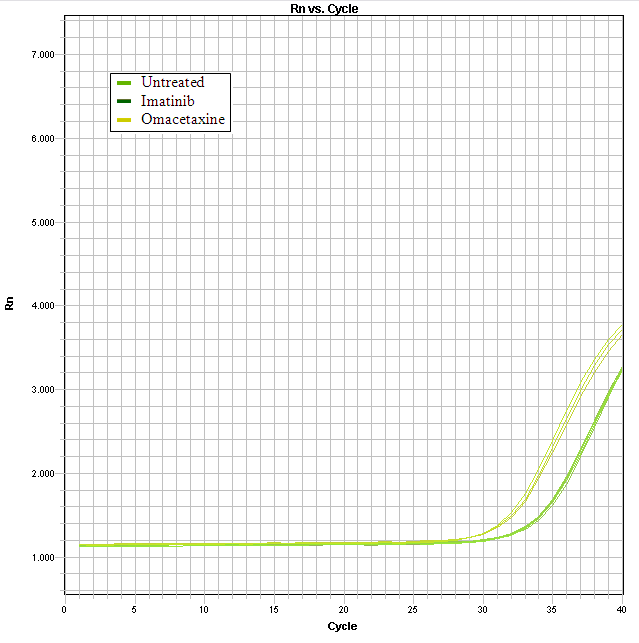

Supplement: Additional file 3 — Figure S3. Representative amplification plots from RT-PCR for selected genes. [file 1755-8794-5-37-S3.doc]
