# Supplementary material for: Association of differential gene expression with imatinib mesylate and omacetaxine mepesuccinate toxicity in lymphoblastoid cell lines
Source: BMC Med Genomics. 2012 Aug 23;5:37. doi: 10.1186/1755-8794-5-37 (PMC3483163; doi:10.1186/1755-8794-5-37)
Supplement: Additional file 4 — Table S2. Gene-specific correlation between the results of q-PCR and microarray. [file 1755-8794-5-37-S4.doc]

**Additional File 4:**

**Supplementary Table 2**

**Gene-specific correlation between the results of qPCR and microarray**

| **Gene** | **Imatinib response** | | **Omacetaxine response** | |
| --- | --- | --- | --- | --- |
|  | **Spearman’s ρ** | **P** | **Spearman’s ρ** | **P** |
| *TNFAIP3* | 0.3849 | 0.0063 | 0.1657 | 0.2014 |
| *OIP5* | 0.0117 | 0.9370 | 0.2014 | 0.2188 |
| *MUL1* | 0.2609 | 0.0733 | 0.2620 | 0.0720 |
| *CTSB* | 0.4086 | 0.0039 | 0.6494 | <0.0001 |
| *BCL2L10* | 0.3366 | 0.0193 | 0.1583 | 0.2879 |
